# Impacts of alfalfa nutrient concentrate on pellet physical attributes, growth performance, metabolism and nutritional quality of rainbow trout, *Oncorhynchus mykiss*

**DOI:** 10.1016/j.aninu.2024.07.011

**Published:** 2024-11-08

**Authors:** Hu Chen, Patrick C. Blaufuss, Dong-Fang Deng, Fabio Casu, Emma K. Kraco, Brian Shepherd, Wendy M. Sealey, Aaron M. Watson, Matthew F. Digman, Deborah A. Samac

**Affiliations:** a School of Freshwater Sciences, University of Wisconsin, Milwaukee, WI 53204, USA; b National Institute of Standards and Technology (NIST), Charleston, SC 29412, USA; c South Carolina Department of Natural Resources, Marine Resources Research Institute, Charleston, SC 29412, USA; d USDA/ARS/University of Wisconsin, Milwaukee, WI 53204, USA; e USDA/ARS Bozeman Fish Technology Center, Bozeman, MT 59715, USA; f Biological Systems Engineering, University of Wisconsin, Madison, WI 53706, USA; g USDA/ARS, Plant Science Research Unit, St. Paul, Minnesota 55108, USA

**Keywords:** Alfalfa, Alternative ingredient, Pellet physical quality, Nutrient metabolism, Rainbow trout

## Abstract

This study addressed the escalating demand for aquatic feed by exploring the potential of alfalfa nutrient concentrate (ANC) as feed ingredient for rainbow trout. Test diets contained varying ANC levels (0%, 5%, 10%, 15%, and 20%) to replace fishmeal (32% in the 0% ANC diet) to achieve equal digestible protein and were processed using a cooking extrusion method. Analysis of feed pellets showed that pellet density increased with ANC levels (*P* < 0.001), resulting in sinking pellets at 20% ANC. Water stability and durability were improved while oil leakage decreased with increasing ANC levels (*P* < 0.05). Two feeding trials were conducted to test the diets in flow-through water systems with three replicates per diet. The first 10-week trial evaluated their impact on feeding, fecal physical quality, and the apparent digestibility coefficient (ADC) of dietary nutrients in rainbow trout (initial body weight 18.0 ± 0.2 g). ANC inclusion did not impact the palatability and satiety feed intake of the fish (*P* > 0.05). However, the ADC of dry matter and phosphorus significantly decreased in fish fed the 20% ANC diet (*P* < 0.05). The second 9-week trial investigated the growth performance, nutrition quality, and metabolism of rainbow trout (initial body weight 19.0 ± 0.2 g). While all fish exhibited substantial growth, fish fed diets with 10% to 20% ANC displayed lower specific growth rate and higher feed conversion ratio compared to those fed with 0% or 5% ANC (*P* < 0.05). The whole body protein content was higher in fish fed 5% ANC compared to all other treatments (*P* = 0.030). The biochemical parameters of plasma were similar across treatments, except for a decrease in plasma phosphorus levels in fish fed a 10% ANC diet compared to those fed a 0% ANC diet (*P* = 0.033). Significant changes were observed in liver metabolism including tricarboxylic acid cycle, amino acid and energy metabolism pathways in fish fed the 20% ANC diet versus the 0% ANC diet (*P* < 0.05). These results demonstrate that ANC inclusion improved pellet physical quality without impairing feeding behavior and nutritional quality of the fish but inclusion ≥10% in the diet reduced fish growth. This study offers the first comprehensive assessment of the potential of ANC used in fish feed involving feed management, feeding evaluation, and the biological response.

## 1 Introduction

Aquaculture plays a crucial role in meeting the escalating global demand for food, as it reached 130.9 million tonnes attributed to 59% of global fisheries and aquaculture production (FAO, 2024). As wild fish stocks reach a plateau, the significance of aquaculture in satisfying seafood consumption is expected to grow further. However, the sustainable expansion of the aquaculture industry encounters challenges, particularly concerning the environmental impact and sustainability of feeds and feed ingredients. Traditionally, fishmeal has been the preferred protein and lipid source in aquaculture feeds due to its optimal nutritional profile. Nevertheless, limited availability and supply variability have resulted in rising prices and instability in supply (Jones et al., 2020). To address this issue, extensive efforts have been made to explore alternative ingredients for aquatic feed production, including plant seed meals, animal by-products, insect meals, and even plant foliage (Dorothy et al., 2018; Jirsa et al., 2014; Jones et al., 2020; Kiron et al., 2016; Nogales-Mérida et al., 2018). One such alternative ingredient under investigation is alfalfa nutrient concentrate (ANC) made from alfalfa leaf, which is recognized for its high protein production per acre of land and widespread use as a feed source for dairy and beef producers (Bals et al., 2012; Radovic et al., 2009). The original whole crop's nutrient composition isn't suitable for aquatic feed due to its low protein concentration (18%) and high fiber content (23%) (Ali et al., 2003; Fernandez et al., 2019). Hence, processing is necessary to enhance its nutrient profile. ANC, derived from alfalfa leaf juice, improves protein content up to 50% while reducing indigestible nutrients like crude fiber, acid detergent fiber, and phytic acid. While ANC has demonstrated benefits such as enhanced feed efficiency, improved meat and egg quality, and increased animal health parameters in poultry and swine diets (Gaweł and Grzelak, 2012), its evaluation as an aquatic feed ingredient is limited. Studies examining ANC as a protein substitute in fish feed have yielded mixed results, suggesting potential efficacy variations across different fish species (Chatzifotis et al., 2006; Coburn et al., 2021; Olasunkanmi et al., 2021). Olvera-Novoa et al. (1990) reported that weight gain, specific growth rate, feed intake and nitrogen deposition in *Oreochromis mossambicus* were the highest when 15% to 20% of fishmeal protein was replaced by alfalfa protein inclusion. However, in a previous study focusing on rainbow trout feed supplementation with 3% or 6% ANC to replace fishmeal protein didn't significantly impact fish growth performance compared to a control diet (Coburn et al., 2021). However, the inclusion levels weren't sufficient to fully evaluate this ingredient's potential. Conversely, when 18% ANC replaced fishmeal in yellow perch feed, fish growth decreased, though no adverse effects on fish feeding were observed. Thus, in our current study, we aimed to exam in a range of ANC inclusion levels from 0% to 20% in rainbow trout feed to test the potential of this ingredient.

Most existing studies on aquatic feed ingredients have primarily focused on cold extruded feeds, which may not fully represent the qualities of feeds produced using cooked extruding commonly employed in the industry. Cooked extruding involves high temperatures and pressures and could significantly enhances both the physical attributes (e.g., water stability) and nutritional qualities (e.g., digestibility) of the feed, rendering it more suitable to the industry. Therefore, studies utilizing this method can provide more practical results for the industry. Furthermore, a comprehensive evaluation of ingredient potential requires a holistic approach encompassing ingredient characterization, impact on palatability, nutrient digestibility and utilization, as well as pellet characteristics (Glencross, 2020; Glencross et al., 2007). Consequently, the present study aims to address this knowledge gap by investigating the effects of ANC in feed for rainbow trout (*Oncorhynchus mykiss*), an important carnivorous fish species in aquaculture. Rainbow trout is one of the few aquaculture species for which we possess a comprehensive understanding of its nutrient requirements (NRC, 2011), making it easier to develop a basic feed formulation for testing the potential of new ingredients. In this study, we assessed the quality of ANC by examining its influence on feed management, palatability, nutrient digestibility and fecal quality, liver metabolism, and overall nutritional quality of rainbow trout. The objective is to provide baseline information for developing aquaculture feeds incorporating ANC.

## 2 Materials and methods

### 2.1 Animal ethics statement

The experimental management for both trials followed the protocols (19-20#11) approved by the Institutional Animal Care and Use Committee of the University of Wisconsin-Milwaukee, USA.

### 2.2 Diet formulation and processing

Five test diets were formulated to include different levels (0%, 5%, 10%, 15%, and 20%) of ANC (Vitalfa LLC, 5555 Brooks Street, Montclair, California, USA) to replace menhaden fishmeal protein at an equivalent amount of digestible protein (Table 1). The test diets were formulated based on available nutrient requirements of rainbow trout and variations for inclusion of plant source proteins (Barrows et al., 2010, 2008; NRC, 2011). Digestible protein levels were estimated based on the apparent digestibility coefficients (ADC) of 77% and 65% for menhaden fishmeal and ANC, respectively, previously determined by the Bozeman Fish Technology Center (Bozeman, Montana, USA; data not published). Yttrium oxide was added to the feed as an inert marker for measuring ADC. Test diets were manufactured at the Bozeman Fish Technology Center via heated extrusion (DNDL-44, Buhler AG, Uzwil, Switzerland). Mixed ingredient mash was exposed for 20 s to an average of 122 °C in the sixth extruder barrel section. The die plate was heated to an average temperature of 90 °C and pressure at the die head was maintained at 35 to 50 bar depending on the test diet formulation. Feed pellets of approximately 2.5 mm diameter were produced and dried in a pulse-bed drier (Buhler AG, Uzwil, Switzerland) for 25 min at 102 °C, followed by cooling on a forced air-cooling table until reaching a final moisture level of less than 7%. Pellets were then top coated with additional fish oil and soybean oil based on the feed formulation using a vacuum coater (A.J. Mixing, Ontario, Canada). Test diets were delivered to the University of Wisconsin-Milwaukee and stored at −20 °C until use. The analytical methods for nutrient compositions were described in section 2.5 of this paper and results are presented in Table 1, Table 2, Table 3.

**Table 1:** Feed formulation and the proximate composition of test diets, FM, and ANC^1^.

Item	ANC-0	ANC-5	ANC-10	ANC-15	ANC-20	FM	ANC
**Ingredients, g/kg**							
Menhaden fish meal	320.0	291.0	262.2	233.2	204.5		
Corn protein concentrate	60.0	60.0	60.0	60.0	60.0		
Soy protein concentrate	160.0	160.0	160.0	160.0	160.0		
Poultry blood meal	80.0	80.0	80.0	80.0	80.0		
Menhaden fish oil	90.0	92.4	94.6	96.9	99.1		
Soybean oil	30.0	23.6	17.1	10.7	4.3		
Wheat gluten meal		1.1	5.5	8.4	11.3		
Alfalfa nutrient concentrate		50.0	100.0	150.0	200.0		
Wheat flour	164.1	143.6	120.5	98.9	77.1		
Lecithin	10.0	10.0	10.0	10.0	10.0		
Stay-C35^2^	3.0	3.0	3.0	3.0	3.0		
Vitamin premix^3^	10.0	10.0	10.0	10.0	10.0		
Sodium chloride	2.8	2.8	2.8	2.8	2.8		
Magnesium oxide	0.6	0.6	0.6	0.6	0.6		
Potassium chloride	5.6	5.6	5.6	5.6	5.6		
Monocalcium phosphate	20.0	22.0	23.5	25.0	26.6		
Choline chloride	10.0	10.0	10.0	10.0	10.0		
DL-Methionine	3.2	3.4	3.5	3.6	3.7		
Lysine hydrochloride	12.7	12.8	12.9	13.0	13.0		
Threonine	1.0	1.1	1.2	1.3	1.4		
Taurine	5.0	5.0	5.0	5.0	5.0		
Mineral premix^4^	1.0	1.0	1.0	1.0	1.0		
Yttrium oxide	1.0	1.0	1.0	1.0	1.0		
Grobiotic A^5^	10.0	10.0	10.0	10.0	10.0		
Total	1000.0	1000.0	1000.0	1000.0	1000.0		
**Nutrient levels, g/kg as fed**							
Moisture	42.6	41.6	44.2	43.6	44.4	80	78
Crude protein	508	514	525	520	530	645	522
Crude lipid	149	149	137	147	138	94	84
Ash	102	106	113	116	118	190	99
Crude fiber	3.8	4.9	6.0	17.3	17.3	0	27

ANC = alfalfa nutrient concentrate; FM = fish meal.

1 ANC-0, ANC-5, ANC-10, ANC-15, and ANC-20 indicate the test diets containing 0%, 5%, 10%, 15%, or 20% ANC, respectively, to replace fishmeal of the test diets.

2 Stay-C35, DSM-Firmenich, New Ulm, Minnesota 56073-160, USA.

3 Vitamin mixture: vitamin A palmitate 965 IU, cholecalciferol 640 IU, D-alpha vitamin E 13.2 IU, menadione sodium bisulfite 0.470 mg, thiamine mononitrate 0.910 mg, riboflavin 0.960 mg, pyridoxine HCl 1.370 mg, DL-pantothenate calcium, 10.110 mg, cyanocobalamin 0.003 mg, nicotinic acid 2.180 mg, biotin 0.033 mg, folic acid 0.250 mg. Wheat flour was added to bring it up to 1 g.

4 Mineral premix (g/kg premix): CaCO_3_ 349.18, CuSO_4_·5H_2_O 59.00, FeSO_4_·7H_2_O 398.50, MnSO_4_·H_2_O 52.60, KI 7.86, Na_2_SeO_3_ 0.96, ZnSO_4_7H_2_O 131.90.

5 Grobiotic A, International Ingredient Corporation, San Louise, MO, USA.

**Table 2:** Mineral content of FM, ANC and the test diets fed to rainbow trout^1^.

Item	ANC-0	ANC-5	ANC-10	ANC-15	ANC-20	FM	ANC
**Macro-minerals, g/kg as fed**							
Sulfur	7.6	7.5	7.7	7.7	7.5	8.2	5.5
Phosphorus	16.4	16.5	16.9	16.9	16.6	43.0	14.6
Potassium	10.6	10.8	11.3	11.3	10.9	0.7	0.9
Magnesium	1.9	2.0	2.1	2.2	2.2	22.0	1.7
Calcium	20.0	21.2	23.3	24.8	25.7	41.2	26.9
Sodium	4.3	4.2	4.1	3.9	3.6	0.4	0.03
**Trace minerals, mg/kg as fed**							
Iron	756	813	911	969	1010	550	1100
Manganese	44.0	49.9	56.4	62.6	66.8	40	75
Copper	38.3	39.5	40.9	43.4	42.4	10	11
Zinc	96.1	91.9	91.3	90.4	89.6	79	18

ANC = alfalfa nutrient concentrate; FM = fish meal.

1 ANC-0, ANC-5, ANC-10, ANC-15, and ANC-20 indicate the test diets containing 0%, 5%, 10%, 15%, or 20% ANC, respectively, to replace fishmeal of the test diets.

**Table 3:** Amino acid profiles of FM, ANC and the test diets fed to rainbow trout^1^.

Item	ANC-0	ANC-5	ANC-10	ANC-15	ANC-20	FM	ANC
**Indispensable amino acids, g/kg ingredient or diet as fed**							
Arginine	29.4	31.7	32.4	27.9	30.9	36.6	31.3
Histidine	18.0	16.8	16.4	16.0	15.9	17.8	13.2
Isoleucine	21.7	19.6	19.8	19.8	21.2	25.7	26.3
Leucine	51.7	50.6	52.6	48.6	46.4	45.4	46.2
Lysine	47.2	45.7	45.9	42.2	42.4	48.1	31.5
Methionine	10.3	10.6	10.0	9.8	10.1	21.6	10.7
Phenylalanine	28.3	26.6	28.6	27.7	30.6	25.1	31.1
Threonine	19.5	19.8	19.6	19.9	21.4	31.7	29.0
Tryptophan	8.2	8.6	8.6	8.4	8.6	66.0	80.0
Valine	28.8	28.4	29.7	27.6	30.3	30.3	31.2
**Dispensable amino acids, g/kg ingredient or diet as fed**							
Alanine	35.0	31.2	32.2	33.5	35.1	46.9	36.0
Asparagine	52.4	49.5	50.6	50.0	51.6	67.2	58.8
Cysteine	5.7	5.4	5.9	5.1	5.4	5.3	3.4
Glutamic acid	89.1	77.8	78.4	80.3	77.7	101.4	63.0
Glycine	35.2	26.9	27.3	28.1	29.1	55.3	33.1
Proline	31.9	28.6	29.4	29.3	31.2	31.6	16.6
Serine	25.8	23.2	24.5	24.1	25.8	29.5	26.3
Tyrosine	18.9	17.9	19.2	17.3	19.3	23.2	26.0

ANC = alfalfa nutrient concentrate; FM = fish meal.

1 ANC-0, ANC-5, ANC-10, ANC-15, and ANC-20 indicate the test diets containing 0%, 5%, 10%, 15%, or 20% ANC, respectively, to replace fishmeal of the test diets.

### 2.3 Physical properties of test diets

The physical properties of feed pellets evaluated in this study included bulk density, water stability, durability index, sinking rate, and oil retention capacity. Each diet was tested in triplicate. For bulk density, a 250-mL container was filled with pellets while gently stacked and then weighed using an analytical balance. For water stability, two grams of pellets were soaked in 150 g distilled water at room temperature (24 °C) for either 15 or 30 min, followed by filtration through a 1 mm mesh filter. The retained pellets were collected and dried at 105 °C for 24 h to obtain the dry weight of the retained pellets. Pellet durability was determined following the method described by Hauptman et al. (2014) using NHP100 portable pellet durability tester (Holmen, Norfolk, UK). Feed pellets (50 g) tumbled for 60 s in the test chamber and remaining sample pellets were weighted. The pellet durability index (PDI) was the difference between pellet weight before and after the test expressed as a percentage of initial weight. For pellet sinking rate, one hundred pellets were added into a glass beaker (18 cm height) filled with 3 L of water. The number of pellets remaining on the water surface was recorded every 5 min over a 90-min period to obtain the percentage and rate of feed pellets sinking. For oil leakage, 10 g of feed pellets were weighed and placed onto absorbent paper in an aluminum pan. The pans were left at 40 °C for 24 h and then cooled to ambient temperature. The feed pellets were removed and the aluminum pan with the absorbent paper was weighed. The following equations were used for the calculations:

Durability index (%) = 100 × pellet weight after tumbling (g)/pellet weight before tumbling (g);

Bulk density (g/L) = pellet weight (g)/a given volume of the pellets (L);

Oil leaking (%) = 100 × fat content loss in the absorbent paper (g)/fat content of feed pellets (g);

Water stability (%) = 100 × dry pellet retention weight (g) during a given period/original pellet weight (g).

### 2.4 Fish source

Rainbow trout fingerlings (0.4 g) were transported from a commercial hatchery to the School of Freshwater Sciences and were quarantined indoor with a control photoperiod (12 h:12 h = light:dark) in a flow through system with water temperature at 14 °C for four weeks before being transferred to the testing laboratory. Juveniles were fed with a commercial feed (45% protein and 15% lipid, Skretting, UT, USA) until reaching the size needed for each experiment.

### 2.5 Feeding trial 1: Palatability, satiety feeding, fecal particle distribution, and digestibility

#### 2.5.1 Fish maintenance

Before feeding trial 1, juveniles were selected and acclimated to a culture system with flow-through water (3 L/min per tank) for two weeks. After two weeks, a total of 300 fish were mixed into a 5-foot tank and then 10 uniform fish (18.2 ± 0.16 g, *n* = 10) were collected and stocked into round tanks (0.58 m diameter × 0.56 m height, 100 L water). Three tanks were randomly assigned to each test diet. During the experimental period, fish were hand-fed designated diets to satiation twice daily at 10:00 and 16:00 except for adjustments needed for sample collections as described below. The total weight of fish for each tank were weighed every two weeks to monitor their growth. The feeding rate ranged from 2.5% to 4% of body weight (BW) during the feeding trial. Water temperature and dissolved oxygen were monitored daily and maintained at 13 to 15 °C and 7 mg/L, respectively. Ammonia and pH were checked weekly and were maintained at less than 0.02 mg/L for ammonia and pH at 7.0 to 8.0 for the duration of the feeding trial. Photoperiod was maintained at 12 h:12 h = light: dark.

#### 2.5.2 Palatability

Palatability was measured during the second week of the feeding trial. The feed were hand-fed at 10:00 and fish were allowed to feed until fish displayed no attempts to feed after 3 s. The remaining feed was removed from the tank and then dried at 105 °C for 24 h to obtain the dry weight. Palatability was estimated based on total feed intake (% BW) and feeding time, active feeding duration, and feed intake (% BW) during the time frame of active feeding.

For the second feeding in the afternoon at 16:00, fish were fed using automatic feeders. The feed loaded into the feeder was based on 1.5% of the total biomass of fish in each tank. This feeding level exceeded satiation for the fish, ensuring that all fish were maintained at satiation feeding in the afternoon. This approach aimed to minimize any confounding effects on the palatability test conducted the following morning. The same procedure was repeated for five days.

#### 2.5.3 Fecal particle distribution

Fecal samples were collected for the measurement of fecal particle distribution during the seventh week of the feeding trial. All tanks were thoroughly siphoned to remove all waste in the tanks 30 min before feeding at 10:00. Four hours post-feeding, feces were siphoned and filtered through nylon nets of different mesh sizes (1000, 250, and 100 μm). All nylon nets were pre-labeled and weighed with a reading accuracy of the nearest 0.01 g. Nylon nets with fecal samples were stored at −20 °C until analysis. Water collected from each filtration was weighed, and a sub-sample of 100 g was transferred to a pre-weighed aluminum pan and dried in an oven at 85 °C and then 100 °C until all water evaporated. The dry samples were weighed and stored at −20 °C until analysis. The same procedure was repeated for four days, and samples collected from each tank were combined for analysis.

Particle distribution (%) = 100 × fecal weight of each size class (g DM)/total fecal weight (g DM).

#### 2.5.4 Satiety feeding

Satiety feeding was conducted during the eighth week of the feeding trial. Pre-weighed feed was hand fed to the fish at 10:00 and 16:00, and the fish were allowed to feed for 30 min. All left-over feed pellets were removed from each tank and dried at 105 °C for 24 h. The same protocol was repeated twice daily for three days. Total feed intake was calculated as:

Feed intake (% BW) = 100 × [dispensed feed (g DM) - leftover feed (g DM)]/BW (g).

#### 2.5.5 Digestibility

Fish continued to be fed to satiation until the end of the tenth week of the feeding trial, at which point fecal samples were collected by manual stripping 16 to 18 h post-feeding. All fish in each tank were anesthetized with MS-222 (75 mg/L), followed by gently drying and then applying pressure to the lower abdominal region to express fecal matter. The fish were returned to their original tanks for recovery, with feeding resuming the next day. The fecal collection was repeated 7 times, with the interval between each sampling being 2 to 4 days. Fecal samples from a given tank were pooled, freeze-dried, and stored at −20 °C until analysis. ADC were calculated using the formula:

ADC (%) = 100 × [1 − (yttrium in feed × nutrient in feces)/(yttrium in feces × nutrient in feed)].

### 2.6 Feeding trial 2: Growth performance, fish health and metabolism

#### 2.6.1 Fish maintenance

Feeding trial 2 was conducted in a system supplied with flow-through water at a rate of 4 L/min per tank (1.0 m diameter × 0.71 m height, 250 L water). Each tank was cleaned and siphoned daily. Mortality, water temperature, pH, and dissolved oxygen were monitored daily, while total ammonia nitrogen was monitored weekly. Water temperature and dissolved oxygen were maintained at 15 to 17 °C and higher than 7.0 mg/L, respectively (YSI Pro1020, YSI Life Sciences, Yellow Springs, Ohio, USA)). Total ammonia nitrogen and pH were measured using a test kit (Ammonia Test Strips, Hach, Loveland, CO, USA) and a pH meter (YSI Pro 1020). The ammonia nitrogen was maintained at levels lower than 0.02 mg/L. The pH was in the range of 7.2 to 8.1. The photoperiod was maintained at 12 h:12 h = light: dark.

Fish were acclimated to the system for two weeks and fed with a commercial feed (45% protein and 15% lipid, Skretting USA) during the first week. At the end of the acclimation period, all fish were fasted for 24 h and then pooled into a large tank for selection and redistribution. Thirty fish (19.0 ± 0.2 g, *n* = 15) were randomly distributed into each tank with three tanks for each experimental diet. Fish were fed using automatic feeders four times daily (09:00, 12:00, 15:00, and 17:00) based on estimated feeding rates. Fish were batch-weighed at every three-week interval, and the feeding rate was adjusted accordingly. During the feeding trial the feeding rate ranged from 2.5% to 5.0% of BW.

#### 2.6.2 Sample collections

At the end of the 9-week feeding trial, all fish of each tank were fasted for 24 h before being batch-weighed and counted. Four fish from each tank were randomly collected, euthanized by buffered MS-222 (300 mg/L, Sigma-Aldrich, USA), and kept at −80 °C for final whole-body proximate composition analysis (moisture, crude protein, crude lipid, and ash). Four fish per tank were randomly selected and then euthanatized for individual BW and total length to calculate the condition factor (CF). They were then dissected to obtain liver, intestine, viscera fat, and carcass for the calculations of hepatosomatic index (HSI), intestine length to body length ratio, visceral fat index (VFI), and carcass index (CSI), respectively. Liver tissues were frozen in liquid nitrogen and stored at −80 °C until analysis. An additional four fish from each tank were anesthetized MS-222 (100 mg/L) to collect blood via caudal puncture using 1.5-mL heparinized syringes and then euthanized with MS-222 (300 mg/L) before liver tissues were collected for metabolomic analysis. Blood samples were set on ice for 4 h and then centrifuged at 4000 × *g* for 20 min at 4 °C. The plasma supernatant was frozen at −80 °C until analysis for biochemical parameters.

#### 2.6.3 Sample analysis

Proximate composition analysis of experimental diets and fish samples was conducted following the methods of AOAC (2019). Moisture content was measured by pulverizing whole fish and drying it in a vacuum freeze dryer for 48 h to reduce the moisture content below 10%, with subsamples being further dried in an oven at 105 °C for 12 h or until a constant weight was achieved (AOAC method 950.46). Crude protein content was determined by measuring total nitrogen (N × 6.25) levels using an elemental combustion system (ECS 4010 nitrogen/protein analyzer, Costech Analytical Technologies Inc., USA; AOAC method 990.03). Crude lipid content in the feed was determined post acid hydrolysis. The crude lipid content of hydrolyzed feed samples and whole fish was subsequently obtained by ether extraction (AOAC method 2003.05) using a Soxhlet extractor (Soxtec 8000, Foss, Denmark). Ash content was determined (AOAC method 942.05) using a muffle furnace at 550 °C for 12 h. Crude fiber was determined following method AOAC method 978.10. Minerals of fecal samples were analyzed using Inductively Coupled Plasma Mass Spectrophotometry (Thermo Jarrell-Ash IRIS Axial ICPOES, Thermo Fisher Scientific, Massachusetts, USA). Minerals and amino acids of feed were analyzed by Midwest laboratories (Omaha, Nebraska 68144, USA) following the methods of AOAC method 985.01 and AOAC method 994.12, respectively.

Metabolomic analysis was performed at the Hollings Marine Laboratory (Charleston, South Carolina, USA) following a similar protocol to the one described by Lu et al. (2022). Liver samples were pulverized in liquid nitrogen using a freezer/mill (Model 6775, SPEX SamplePrep, USA). Samples were weighed (100 ± 3 mg) into 2 mL metal bead tubes (2.38 mm, Qiagen, USA). A modified version of the solvent extraction technique by (Bligh and Dyer, 1959) was used as described by (Casu et al., 2017). Pulverized tissue was first homogenized in ice-cold methanol and Millipore ultrapure 18.2 MΩ water using a bead beater (2 cycles at 6500 r/min for 15 s/cycle) (Precellys 24, Bertin Corp., Rockville, MD, USA). The polar homogenates were then mixed with chloroform and Millipore deionized water to reach a final volume ratio of 2:2:1.8 chloroform: methanol: water. The mixture was vortexed and then centrifuged at 2000 × *g* for 5 min at 4 °C. The top polar layer was isolated and dried, followed by reconstitution of the dried metabolites with NMR buffer (100 mmol/L phosphate buffer in D_2_O, pH 7.3, with 1.0 mmol/L sodium 3-(trimethylsilyl) propionate 2,2′,3,3′-d (TMSP)). The resulting solution was centrifuged, and the supernatant was transferred into 5 mm NMR tubes (Bruker Biospin Inc., USA) for NMR analysis (Casu et al., 2017). Spectra were acquired using an automated SampleJet sample changer on a Bruker Avance II 700 MHz NMR spectrometer equipped with a 5 mm triple-resonance, z-gradient TCI cryoprobe at 298 K. Spectra acquisition and processing was performed as described in Lu et al. (2022).

### 2.7 Data analysis and statistics

All data except for the metabolomic data were checked for normality and homogeneity before analysis with Shapiro-Wilk and Levene tests, respectively. All data were presented at mean ± standard error (*n* = 3) were evaluated by one-way ANOVA with Tukey's HSD as the post hoc test using Statistical 13.0 (StatSoft, USA), with differences considered significant at *P* < 0.05. The mathematical model for one-way ANOVA is expressed as:

*Y*_*ij*_ = *μ* + *α*_*i*_ + *ϵ*_*ij*_*,*

Where *Y*_*ij*_ is the *j*-th observation in the *i*-th group; *μ* is the overall mean of all observations; *α*_*i*_ is the main effect of the *i*-th level (group mean minus overall mean); *ϵ*_*ij*_ is the random error associated with the *j*-th observation in the *i*-th group, representing the influence of other random factors beyond the independent variable on the dependent variable. The mathematical model for polynomial regression is expressed as:

*y* = *β₀* + *β*₁*x* + *β*₂*x*^2^ + *β*₃*x*³ + … + *βₙxⁿ* + *ε*,

where *y* is the dependent variable; *x* is the independent variable; *β*₀, *β*₁, *β*₂, … *βₙ* are the regression coefficients; *ε* is the error term, with *n* representing the order of the polynomial used to fit the regression. The correlation between different responses to dietary ANC levels was determined using a linear or the 2nd order polynomial regression analysis (Zeitoun et al., 1976).

For metabolomic analysis, significant metabolites were identified using both 1D ^1^H NOESY and 2D ^1^H-^13^C HSQC NMR spectra. Chemical shifts were compared with reference spectra in available metabolomic databases including the Human Metabolome Database (HMDB, http://www.hmdb.ca), the Biological Magnetic Resonance Data Bank (BMRB, http://bmrb.wisc.edu/), Chenomx NMR Suite profiling software (version 8.1; Chenomx, Edmonton, Canada), as well as previously published data tables and an in-house compiled database (Nicholson et al., 1995; Ulrich et al., 2008; Wishart et al., 2013). Generally, metabolite identification was achieved at a Level 2-putative identification (Sumner et al., 2007). Spectral features that could not be identified based on database or literature searches were annotated as “Unknown.” Selected spectra were binned with a bin width of 0.005 ppm between *δ* 0.2 ppm to 10.0 ppm as described in (Casu et al., 2019). Specific spectral regions including acetate (1.91 to 1.93 ppm), water (4.70 to 4.90 ppm), chloroform (7.67 to 7.69 ppm) and formate (8.45 to 8.47 ppm) were excluded from the analysis to remove spectral artifacts which were caused by water suppression or contaminants detected in the blank sample spectra. The spectra were then normalized by the total spectral intensity, centered and scaled (Pareto scaling).

For pathway analysis, quantitative Enrichment Analysis (QEA) was performed on a set of 21 metabolites from the dataset of fish fed the ANC-20 diet (*n* = 6) and the ANC-0 diet (*n* = 6) using metabolite set enrichment analysis (MSEA) and the MSEA Metabolic Pathway library (*n* = 88) in MetaboAnalyst 5.0 (Pang et al., 2021). The global test algorithm which uses a generalized linear model to estimate a Q-statistic for each metabolite set was applied. Pathways were considered enriched when *P* < 0.05 and false discovery rate (FDR) <5%. Partial Least Square Discriminant Analysis (PLS-DA) and Orthogonal Partial Least Square Discriminant Analysis (OPLS-DA) were performed to compare the liver metabolite profiles between fish fed the control diet and the diet containing 20% ANC. Data normalization, binning, and spectral alignment were performed using NMRProcFlow 1.3 software (Jacob et al., 2017). Data normalization, centering, scaling and multivariate statistical analysis were performed using MetaboAnalyst 5.0.

## 3 Results

### 3.1 Physical quality of diet pellets

**Fig. 1:**
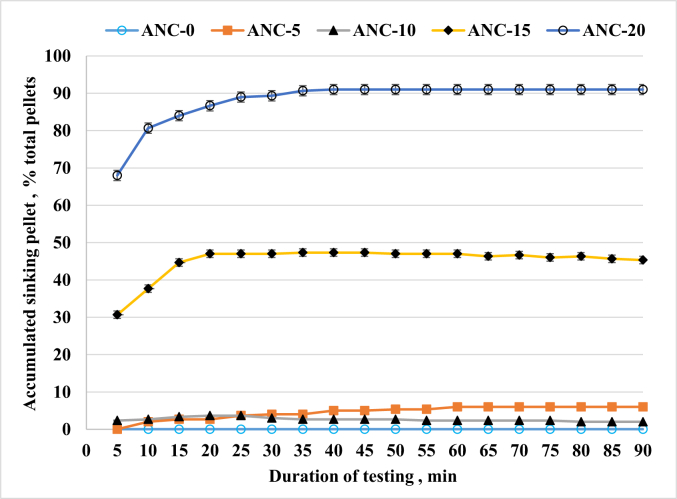
Accumulated sinking pellet (% of total pellets) during a 90-min testing at a water temperature of 14 °C. ANC-0, ANC-5, ANC-10, ANC-15, and ANC-20 indicate the test diets containing 0%, 5%, 10%, 15%, and 20% ANC, respectively, to replace fishmeal of the test diets. Data are presented as the mean of three replicates.

The durability index of pellets showed a significant (*P* < 0.001) increase in the ANC-10, ANC-15, and ANC-20 diets compared to the ANC-0 and ANC-5 diets (Table 4). Additionally, the bulk density of pellets exhibited a significant increase with addition of ANC in the test diets (*P* < 0.001, Table 4). Moreover, the ANC-20 diet resulted in a significant reduction in oil leaking capacity compared to ANC-0 (*P* = 0.010). Furthermore, the water stability at 15 or 30 min was significantly higher for the ANC-10, ANC-15, and ANC-20 diets in comparison to ANC-0 or ANC-5 (*P* < 0.001). While pellets from ANC-0, ANC-5, and ANC-10 diets maintained floating or neutral buoyancy, approximately 50% or 90% of pellets sank within 20 min for the ANC-15 or ANC-20 diets, respectively (Fig. 1).

**Table 4:** Physical properties of test diets for rainbow trout^1^.

Item	ANC-0	ANC-5	ANC-10	ANC-15	ANC-20	*P*-value		
						ANOVA	Linear	Quadratic
Durability index, %	92 ± 0.3^b^	91 ± 0.3^b^	95 ± 0.3^a^	94 ± 0.2^a^	94 ± 0.2^a^	＜0.001	0.009	0.007
Bulk density, g/L	495 ± 2.1^d^	536 ± 3.1^c^	541 ± 0.5^c^	580 ± 2.0^b^	598 ± 1.4^a^	＜0.001	＜0.001	＜0.001
Oil leakage, %	12.1 ± 3.48^a^	7.7 ± 1.31^ab^	6.2 ± 0.34^ab^	1.3 ± 0.35^b^	0.0 ± 0.00^b^	0.010	＜0.001	＜0.001
**Water stability, %**								
15 min	89 ± 0.3^b^	89 ± 0.2^b^	91 ± 0.0^a^	91 ± 0.3^a^	91 ± 0.1^a^	＜0.001	＜0.001	＜0.001
30 min	86 ± 0.1^c^	85 ± 0.1^c^	87 ± 0.0^b^	87 ± 0.8^b^	89 ± 0.1^a^	＜0.001	＜0.001	＜0.001

ANC = alfalfa nutrient concentrate.

^a-d^The different letters within the same row indicate significant differences as tested by Tukey's HSD (*P* < 0.05).

1 ANC-0, ANC-5, ANC-10, ANC-15, and ANC-20 indicate the test diets containing 0%, 5%, 10%, 15%, or 20% ANC, respectively, to replace fishmeal of the test diets. Data are presented as mean ± SE, *n* = 3.

### 3.2 Palatability and digestibility of test diets (Trial 1)

The measurement of feed intake for palatability did not show any significant differences among all dietary treatments (*P* > 0.05; Table 5). However, the active feeding duration varied among the different diets (*P* < 0.001). Fish fed ANC-10 exhibited the longest active feeding duration, while those fed ANC-20 showed the shortest duration. Fish fed the remaining three diets displayed similar active feeding durations. At the end of 8-week feeding, satiety feeding was not different among all the treatments.

**Table 5:** Feed intake and apparent digestibility coefficients of test diets fed to rainbow trout for 8 weeks (Trial 1)^1^.

Item	ANC-0	ANC-5	ANC-10	ANC-15	ANC-20	*P*-value		
						ANOVA	Linear	Quadratic
**Palatability**^2^								
Feed intake, % BW	4.1 ± 0.17	4.3 ± 0.14	4.1 ± 0.04	3.9 ± 0.11	3.8 ± 0.40	0.565	0.124	0.070
Feeding duration, s	109 ± 3.7^b^	102 ± 9.9^bc^	141 ± 5.7^a^	94 ± 4.0^bc^	78 ± 0.1^c^	＜0.001	0.091	0.05
Satiety feeding^3^, % BW	3.2 ± 0.05	3.2 ± 0.09	3.2 ± 0.07	3.4 ± 0.08	3.4 ± 0.08	0.079	0.033	0.030
**Apparent digestibility coefficients, %**								
Dry matter	76 ± 0.5^a^	76 ± 0.5^a^	74 ± 0.8^ab^	73 ± 0.8^ab^	72 ± 0.2^b^	0.007	＜0.001	＜0.001
Crude protein	89 ± 0.4	88.8 ± 0.1	89.3 ± 0.4	89.6 ± 0.5	90.0 ± 0.2	0.105	0.001	0.001
Sulfur	71 ± 0.1^b^	73 ± 1.2^ab^	74 ± 0.2^ab^	74 ± 1.0^ab^	74 ± 0.8^a^	0.048	0.001	0.002
Phosphorus	47 ± 0.1^a^	47 ± 1.4^ab^	44 ± 0.4^bc^	43 ± 1.3^c^	42 ± 0.2^c^	0.002	＜0.001	＜0.001
Magnesium	47 ± 1.0	52 ± 0.8	51.0 ± 2.9	51.0 ± 2.0	52.3 ± 1.4	0.309	0.061	0.040
Calcium	9.3 ± 0.76^ab^	6.2 ± 0.06^b^	7.9 ± 0.86^ab^	9.4 ± 0.80^ab^	10.3 ± 0.07^a^	0.027	0.101	0.004
Iron	5.1 ± 0.69^b^	5.3 ± 1.05^b^	9.0 ± 1.40^ab^	11.3 ± 1.50^a^	12.2 ± 0.45^a^	0.004	＜0.001	＜0.001
Manganese	5.9 ± 0.95	6.5 ± 2.01	7.3 ± 0.86	8.4 ± 1.64	9.8 ± 0.57	0.228	0.008	0.007
Copper	29.3 ± 1.58^c^	36.6 ± 3.86^bc^	44.9 ± 0.91^ab^	47.8 ± 2.55^a^	47.4 ± 0.79^a^	＜0.001	＜0.001	＜0.001
Zinc	24.2 ± 0.81^b^	25.4 ± 1.38^b^	27.2 ± 0.75^ab^	27.7 ± 1.24^ab^	31.0 ± 0.27^a^	0.004	＜0.001	＜0.001

ANC = alfalfa nutrient concentrate; BW = body weight.

^a-c^Different superscript letters between treatments within the same row indicate significant differences as tested by Tukey's HSD (*P* < 0.05).

1 ANC-0, ANC-5, ANC-10, ANC-15, and ANC-20 indicate the test diets containing 0%, 5%, 10%, 15%, or 20% ANC, respectively, to replace fishmeal of the test diets. Data are presented as mean ± SE, *n* = 3.

2 Palatability was determined based on the feed intake of fish measured in the 2nd week of the feeding trial and active feeding duration (s) when the fish were given their first feed of the day.

3 Satiety feeding was determined at the end of 8 weeks of the feeding trial. Fish were allowed to feed to satiation for 30 min and feed intake was measured.

The ADC of dietary crude protein, magnesium, and manganese showed no significant differences among the different treatments (Table 5). However, the ADC of dry matter and phosphorus exhibited a significant decrease with increasing levels of ANC in the diets (*P* < 0.05). In contrast, the ADC of sulfur, iron, copper, and zinc increased as the dietary ANC levels increased (*P* < 0.05). The ADC of calcium, however, significantly decreased when fish were fed a diet containing 5% ANC compared to those on a 0% ANC diet and an increase in ADC was subsequently observed as ANC levels rose from 5% to 20% in the test diets (*P* = 0.027).

### 3.3 Growth performance of fish (Trial 2)

**Fig. 2:**
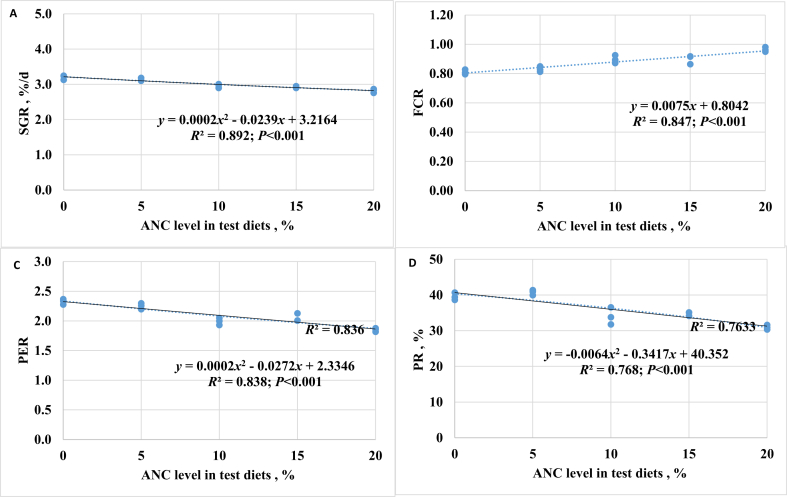
Linear regression analysis of (A) specific growth rate (SGR), (B) feed conversion ratio (FCR), (C) protein efficiency ratio (PER), and (D) protein retention (PR) of rainbow trout in response to feeding with the experimental diets for 9 weeks. ANC = alfalfa nutrient concentrate.

There were significant effects from diet containing 10% to 20% ANC on fish growth performance and feed conversion ratio (FCR), with a significant decrease in growth rate and increased in FCR compared to fish on the ANC-0 and ANC-5 diets (*P* < 0.001; Table 6 and Fig. 2). The inclusion of ANC at or above 10% in the diet led to a significant reduction in protein efficiency ratio (PER, *P* < 0.001) and PR (*P* < 0.001). The growth and PER displayed a negative correlation with the dietary level of ANC. However, the test diets did not have any significant impacts on the morphology of the fish, including CF, CSI, HSI, visceral fat index (VFI), and relative intestine ratio (RIR). Furthermore, similar to the observation in Trial 1, at the end of 9-weeks of feeding, satiety feeding was not significantly influenced by different diets.

**Table 6:** Growth performance of rainbow trout fed test diets for 9 weeks (Trial 2)^1^.

Item^2^	ANC-0	ANC-5	ANC-10	ANC-15	ANC-20	*P*-value		
						ANOVA	Linear	Quadratic
FBW, g	146 ± 2.2^a^	138 ± 2.9^ab^	126 ± 2.9^bc^	119 ± 1.0^cd^	112 ± 4.0^d^	＜0.001	＜0.001	＜0.001
SGR, %/d	3.2 ± 0.04^a^	3.1 ± 0.04^ab^	3.0 ± 0.02^bc^	2.9 ± 0.02^cd^	2.8 ± 0.05^d^	＜0.001	＜0.001	＜0.001
FCR	0.8 ± 0.01^c^	0.8 ± 0.01^c^	0.9 ± 0.02^b^	0.9 ± 0.02^b^	1.0 ± 0.01^a^	＜0.001	＜0.001	＜0.001
CF, g/cm^3^	1.4 ± 0.02	1.4 ± 0.01	1.3 ± 0.02	1.3 ± 0.03	1.4 ± 0.02	0.135	0.459	0.060
CSI, %	88 ± 0.3	89 ± 0.3	88 ± 0.6	89 ± 0.5	88 ± 0.5	0.762	0.576	0.238
HSI, %	1.2 ± 0.02	1.2 ± 0.04	1.2 ± 0.05	1.2 ± 0.04	1.2 ± 0.04	0.835	0.850	0.797
VFI, %	1.5 ± 0.21	1.4 ± 0.26	1.5 ± 0.09	1.6 ± 0.38	1.8 ± 0.24	0.825	0.310	0.197
RIR	0.5 ± 0.03	0.6 ± 0.02	0.5 ± 0.02	0.6 ± 0.01	0.6 ± 0.03	0.429	0.050	0.049
PER	2.3 ± 0.03^a^	2.3 ± 0.03^a^	2.0 ± 0.03^bc^	2.0 ± 0.04^b^	1.9 ± 0.02^c^	＜0.001	＜0.001	＜0.001
PR, %	40 ± 0.6^a^	41 ± 0.8^a^	34 ± 1.4^bc^	35 ± 0.3^b^	31 ± 0.3^b^	＜0.001	＜0.001	＜0.001
Satiety feeding^3^, % BW	3.4 ± 0.13	3.8 ± 0.20	3.1 ± 0.31	3.2 ± 0.17	3.7 ± 0.41	0.411	0.944	0.509

FBW = final body weight; SGR = specific growth rate; FCR = feed conversion ratio; CF = condition factor; CSI = carcass index; HSI = hepatosomatic index; VFI = visceral fat index; RIR = relative intestine ratio; PR = protein retention; PER = protein efficiency ratio; BW = body weight.

^a-d^Different superscript letters between treatments within the same row indicate significant differences as tested by Tukey's HSD (*P* < 0.05).

1 ANC-0, ANC-5, ANC-10, ANC-15, and ANC-20 indicate the test diets containing 0%, 5%, 10%, 15%, or 20% ANC, respectively, to replace fishmeal of the test diets. Data are presented as mean ± SE, *n* = 3. Initial body weight of fish, 19.3 ± 0.20 g, *n* = 15.

2 SGR (%/d) = 100 × ln (final body weight, g/initial body weight, g)/63 days. FCR = (dry feed weight per tank, g)/(total weight gain per tank, g). CF (g/cm^3^) = (body weight, g)/(body length, cm)^3^ × 100. CSI (%) = (carcass weight, g)/(body weight, g) × 100. HSI (%) = (liver weight, g)/(body weight, g). VFI (%) = (visceral fat weight, g)/(body weight, g). RIR, total length of intestine (cm)/full body length (cm). PR (%) = 100 × (final fish body protein, g – initial fish body protein, g)/(protein fed, g). PER = (fish weight gain, g)/(protein fed, g).

3 Satiety feeding (% BW) was determined at the end of 9 weeks of the feeding trial. Fish were allowed to feed to satiation for 30 min and feed intake was measured.

### 3.4 Changes in proximate composition and metabolism of fish (Trial 2)

The proximate compositions of whole fish, liver and muscle tissues did not show significant differences among the dietary treatments, except for the crude protein content in fish fed the ANC-5 diet, which was higher than in the other treatments (*P* = 0.030, Table 7). The crude lipid content of muscle tissues tended to decrease with the increase of ANC supplementation but was not significantly different among treatments. The blood hematocrit and plasma biochemical parameters related to fish health were similar across the different treatments, except for plasma phosphorus concentration, which was significantly lower in the ANC-10 treatment compared to ANC-0 (*P* = 0.033; Table 8).

**Table 7:** Proximate composition (% wet tissue) of rainbow trout fed test diets for 9 weeks (Trial 2)^1^.

Item	ANC-0	ANC-5	ANC-10	ANC-15	ANC-20	*P*-value		
						ANOVA	Linear	Quadratic
**Whole fish**								
Moisture	67 ± 0.2	67 ± 0.2	68 ± 0.5	67 ± 0.1	67 ± 0.4	0.385	0.921	0.145
Crude protein	16 ± 0.2^b^	17 ± 0.1^a^	16 ± 0.4^b^	16 ± 0.1^b^	16 ± 0.1^b^	0.030	0.124	0.087
Crude lipid	12 ± 0.3	12 ± 0.4	11 ± 0.1	12 ± 0.5	13 ± 0.3	0.166	0.683	0.009
Ash	2.0 ± 0.04	2.1 ± 0.06	2.1 ± 0.08	2.2 ± 0.06	2.1 ± 0.04	0.343	0.107	0.056
**Liver**								
Moisture	79 ± 0.2	79 ± 0.6	79 ± 0.1	79 ± 0.5	77 ± 0.5	0.115	＜0.001	0.021
Crude protein	13 ± 0.4	14 ± 0.5	14 ± 0.3	13 ± 0.3	14 ± 0.7	0.303	＜0.001	0.620
Crude lipid	3.6 ± 0.23	4.1 ± 0.41	3.4 ± 0.12	3.8 ± 0.27	4.2 ± 1.04	0.803	0.095	0.453
Glycogen	6.5 ± 0.88	5.7 ± 1.41	3.3 ± 0.29	4.8 ± 0.89	5.8 ± 0.48	0.182	0.486	0.023
**Muscle**								
Moisture	72 ± 0.7	73 ± 0.2	73 ± 0.2	74 ± 0.6	74 ± 0.4	0.056	0.005	0.002
Crude protein	24 ± 0.3	24 ± 0.1	23 ± 0.2	23 ± 0.6	23 ± 0.4	0.103	0.013	0.005
Crude lipid	5.9 ± 0.70	5.4 ± 0.24	5.4 ± 0.37	4.5 ± 0.49	4.9 ± 0.38	0.309	0.051	0.040

ANC = alfalfa nutrient concentrate.

^a,b^Different superscript letters between treatments within the same row indicate significant differences as tested by Tukey's HSD (*P* < 0.05).

1 ANC-0, ANC-5, ANC-10, ANC-15, and ANC-20 indicate the test diets containing 0%, 5%, 10%, 15%, or 20% ANC, respectively, to replace fishmeal of the test diets. Data are presented as mean ± SE, *n* = 3.

**Table 8:** Blood and plasma biochemical parameters from rainbow trout fed test diets for 9 weeks (Trial 2)^1^.

Item	ANC-0	ANC-5	ANC-10	ANC-15	ANC-20	*P*-value		
						ANOVA	Linear	Quadratic
HCT, %	46 ± 0.5	46 ± 0.7	46 ± 1.1	46 ± 2.0	45 ± 1.1	0.954	0.405	0.449
ALB, g/L	29 ± 0.3	27 ± 1.3	26 ± 1.6	26 ± 0.2	29 ± 0.8	0.251	0.625	0.018
ALP, U/L	159 ± 21.8	161 ± 5.3	133 ± 2.7	170 ± 15.6	190 ± 28.2	0.325	0.246	0.056
ALT, U/L	32 ± 6.2	41 ± 6.6	44 ± 6.3	45 ± 15.6	36 ± 8.9	0.847	0.652	0.215
AMY, U/L	172 ± 3.4	166 ± 2.1	146 ± 11.4	162 ± 32.4	129 ± 16.2	0.426	0.096	0.091
CA, mEq/L	6.6 ± 0.07	6.4 ± 0.09	6.5 ± 0.11	6.3 ± 0.07	6.5 ± 0.07	0.449	0.423	0.137
PHOS, mmol/L	6.2 ± 0.17^a^	5.6 ± 0.16^ab^	5.2 ± 0.34^b^	5.9 ± 0.06^ab^	5.9 ± 0.06^ab^	0.033	0.805	0.006
CRE, μmol/L	12.8 ± 1.48	8.3 ± 3.10	11.3 ± 2.43	12.9 ± 1.66	23.2 ± 7.86	0.330	0.078	0.009
GLU, mmol/L	3.8 ± 0.12	3.9 ± 0.08	4.4 ± 0.20	3.7 ± 0.08	4.0 ± 0.24	0.075	0.678	0.758
TP, g/L	45 ± 1.0	44 ± 1.9	42 ± 1.8	45 ± 0.8	44 ± 1.4	0.593	0.680	0.203
GLOB, g/L	17 ± 1.2	17 ± 0.7	16 ± 0.7	18 ± 0.6	16 ± 0.9	0.203	0.767	0.465

ANC = alfalfa nutrient concentrate; HCT = blood hematocrit ALB = albumin; ALP = alkaline phosphatase; ALT = alanine amino transferase; AMY = amylase; CA = calcium; PHOS = inorganic phosphorus; CRE = creatinine; GLU = glucose; TP = total protein; GLOB = globulin.

^a,b^Different superscript letters between treatments within the same row indicate significant differences as tested by Tukey's HSD (*P* < 0.05).

1 ANC-0, ANC-5, ANC-10, ANC-15, and ANC-20 indicate the test diets containing 0%, 5%, 10%, 13%, or 20% ANC, respectively, to replace fishmeal of the test diets. Data are presented as mean ± SE, *n* = 3.

**Fig. 3:**
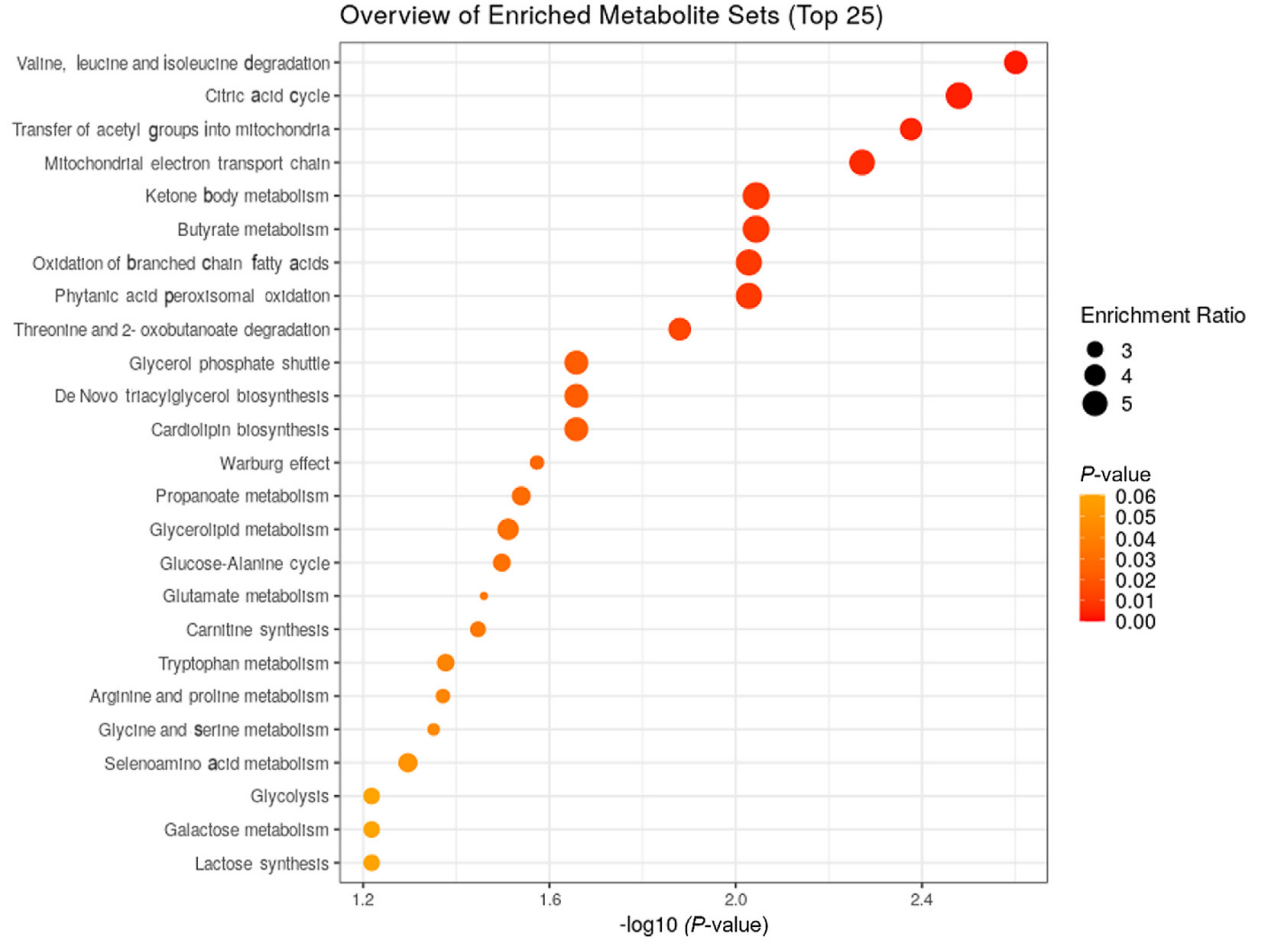
Summary plot for quantitative enrichment analysis (QEA) based on a list of 20 metabolites detected by nuclear magnetic resonance in rainbow trout liver extracts from fish fed the ANC-20 diet compared with fish fed the ANC-0 for 9 weeks. ANC-0 and ANC-20 indicate the test diets containing 0% and 20% ANC, respectively. ANC = alfalfa nutrient concentrate.

**Fig. 4:**
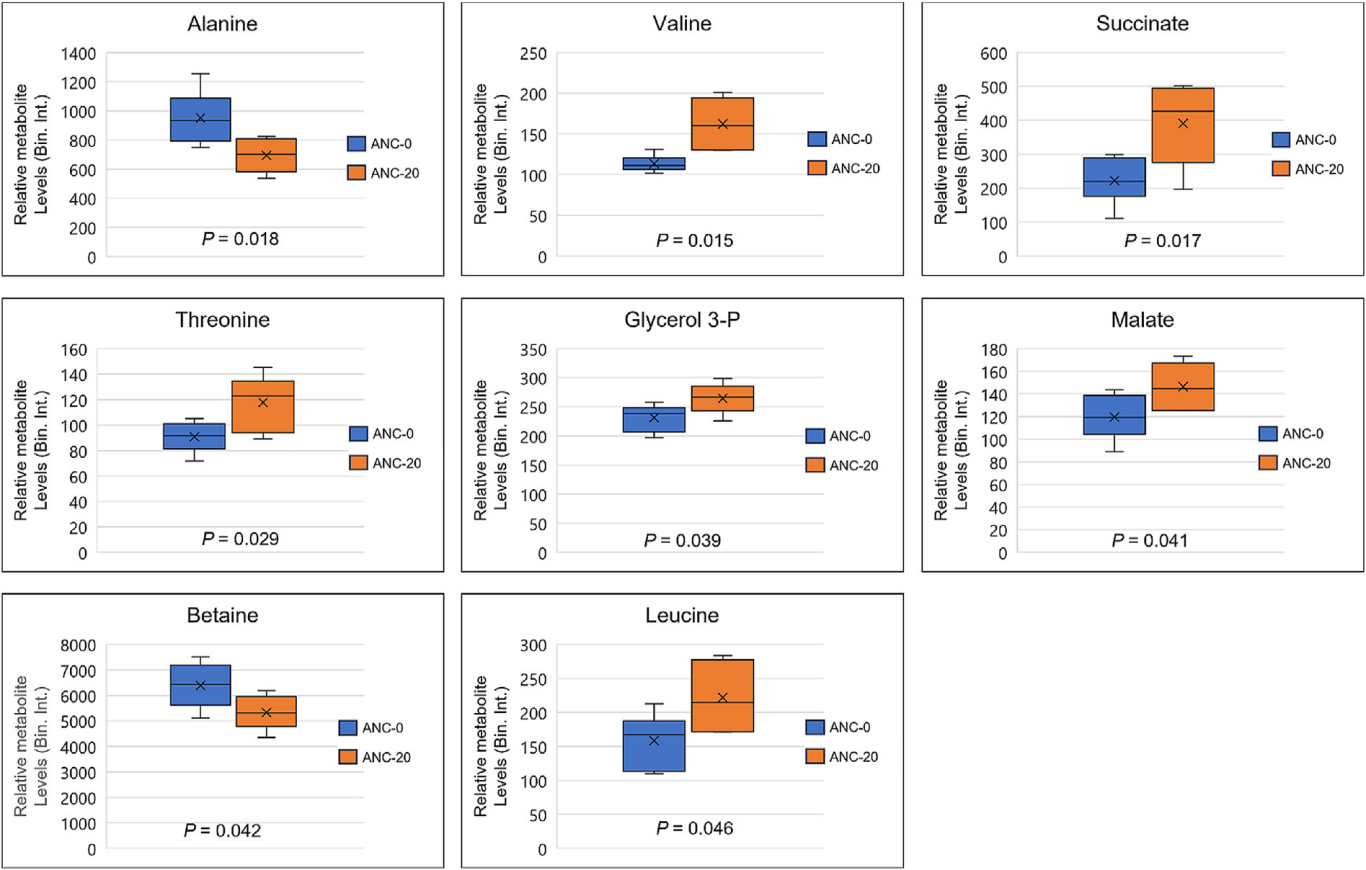
Box plots showing relative metabolite levels for the significantly different metabolites detected in the liver of rainbow trout fed the ANC-0 and ANC-20 diets. ANC-0 and ANC-20 indicate the test diets containing 0% and 20% ANC, respectively. The means from the two groups for each metabolite were compared using *t*-test. *P* < 0.05 indicated significance between the comparison. Glycerol 3-P = glycerol 3-phosphate.

Significant differences were observed in liver metabolites, particularly those involved in amino acid metabolism (alanine, leucine, threonine, valine), citric acid cycle intermediates (succinate, malate), osmolytes (betaine), mitochondrial energy metabolism, ketone body metabolism, and butyrate metabolism, between ANC-0 and ANC-20 (Fig. 3). Specifically, ANC-20 showed significantly lower levels of alanine and betaine compared to ANC-0 (*P* < 0.05), while leucine, valine, threonine, malate, succinate, and glycerol 3-P concentrations were significantly higher in ANC-20 than in ANC-0 (*P* = 0.039; Fig. 4).

### 3.5 Particle size distribution and nutrient content of feces (Trial 1)

The distribution of fecal residuals collected through filters with mesh sizes of 100, 250, and 1000 μm was size-dependent across all dietary treatments. The highest retention was observed with the 1000 μm mesh, followed by the 250 μm and then the 100 μm meshes (Table 9). While the distribution within the 100 to 250 μm range showed no significant differences among dietary treatments, a significant positive correlation was noted for the 250 to 1000 μm range (*P* = 0.036). The distribution of fecal particles from the ANC-0 diet was significantly lower than that of all other treatments. Conversely, fecal particles larger than 1000 μm decreased in samples from fish fed diets with increased level of ANC (*P* = 0.047), with significantly level observed in fish on the ANC-20 diet compared to other treatments. Additionally, the moisture content of feces from fish on ANC-based diets increased (*P* = 0.001), while protein content decreased with higher levels of ANC in the test diets (*P* = 0.000). Fish fed ANC-15 and ANC-20 diets exhibited significantly lower protein content in their feces compared to those fed ANC-0 (Table 9).

**Table 9:** Particle size distribution and moisture content of fecal samples (Trial 1)^1^.

Item	ANC-0	ANC-5	ANC-10	ANC-15	ANC-20	*P-*value		
						Diet	Particle size	Diet × particle size
**Particle distribution**^2^**, %**								
100–250 μm	24.5 ± 1.54^x^	24.5 ± 1.31^x^	23.4 ± 1.16^x^	22.5 ± 0.82^x^	25.7 ± 1.061^x^			
250–1000 μm	33.2 ± 1.56^y^	34.8 ± 1.33^y^	35.0 ± 1.57^y^	35.7 ± 1.77^y^	39.1 ± 1.81^y^	0.998	<0.001	0.054
>1000 μm	42.3 ± 1.69^z^	41.2 ± 2.17^z^	41.7 ± 1.72^z^	41.8 ± 1.07^z^	36.3 ± 0.33^y^			

Item	ANC-0	ANC-5	ANC-10	ANC-15	ANC-20	ANOVA	Linear	Quadratic

Fecal dry matter^3^, %	12 ± 0.5^a^	10 ± 0.5^ab^	9 ± 0.2^b^	9 ± 0.1^b^	8 ± 0.6^b^	0.002	0.001	<0.001
Fecal protein, %	5.6 ± 0.06^a^	5.6 ± 0.10^a^	5.1 ± 0.02^ab^	4.7 ± 0.12^b^	4.7 ± 0.20^b^	<0.001	<0.001	<0.001

ANC = alfalfa nutrient concentrate.

^a,b^Indicate the difference (*P* < 0.05) in fecal particle distribution within the same range across different dietary treatments.

^x,y,z^Indicate the difference (*P* < 0.05) among particle distributions within the same dietary treatment.

1 ANC-0, ANC-5, ANC-10, ANC-15, and ANC-20 indicate the test diets containing 0%, 5%, 10%, 15%, and 20% ANC, respectively, to replace fishmeal of the test diets. Data are presented as mean ± SE, *n* = 3.

2 Particle distribution was measured in newly excreted feces in their designated tanks. The dry matter was measured in feces samples stripped from the fish about 15 h after feeding as described in the method.

3 Fecal dry matter was analyzed for pooled samples of each diet.

## 4 Discussion

### 4.1 The effect of ANC on the physical quality of feed pellets

The findings of this study reveal that diets incorporating ANC contribute to increased pellet durability. This suggests a reduction in breakage during handling or transportation, leading to a decrease in the accumulation of uneaten fine particles within culture systems. While enhanced durability could positively affect feed management, it is crucial to ensure that increased feed durability does not adversely affect palatability and digestibility. Previous research suggested pellet durability may negatively impact fish growth and digestibility (Samuelsen et al., 2021). However, in this study, the durability of feed pellets was not observed to negatively impact the palatability and satiation feeding of rainbow trout, as feed intake was similar across all treatments. The decrease in feeding duration in rainbow trout fed diets ANC-15 or ANC-20 might be attributed to the low buoyancy of these pellets, which were found to sink slowly during the initial 5 min of feeding, allowing fish to catch them more efficiently. Nonetheless, a decline in dry matter digestibility in fish fed the ANC-20 diet suggests that these pellets may be too hard to break down for digestion. Further investigation into this aspect is warranted to determine whether impaired digestibility is due to the physical quality of the feed pellet or an imbalance in nutritional content.

Another improvement in the physical quality of ANC-based feed pellets is the reduction of nutrient leaching, supported by increased water stability and reduced oil leakage. Water stability is crucial for minimizing disintegration and nutrient loss when exposed to water, affecting dietary nutrient availability to the fish and impacting their feeding habits. Previous studies have underscored the significant impact of water stability on feed intake, revealing an increase in rainbow trout and Atlantic salmon when fed low water stability feed (Aas et al., 2011, 2020). Oil leakage not only leads to the loss of dietary fat but can also create an oily film on the surface of a culture system, with potential adverse effects including reduction of oxygen supply to the fish. Although not tested in our current study, previous research has shown that oil leakage from feed pellets can impair the microbiological filters in recirculating aquaculture systems, ultimately reducing water quality treatment efficiency (Chaabani et al., 2020). Moreover, oil leakage can cause changes in pellet density due to the loss of oil and reduced shelf-life of pellets because of the voluntary oxidation of lipids on the pellet surface. Thus, our results suggest that ANC supplementation benefits feeding management, at least in part, by reducing nutrient leaching.

Moreover, pellet density plays a crucial role in determining buoyancy, making it a key factor preferred by different fish species and directly affecting fish feeding efficiency. Effective control of this parameter can be achieved through feed processing management. Additionally, the density of pellets has implications for feed storage capacity; high-density pellets occupy less space compared to low-density pellets containing the same amount of feed nutrients. These findings have practical implications for feed management, encompassing handling, storage, and feeding of feed based on ANC. However, it's worth noting that the density of feed pellets may have varied effects on the digestion capacity of fish. Confirmation of whether this contributes to the observed low digestibility of dry matter in this study requires further research.

### 4.2 The impact of ANC on the physical quality and the nutrient content of feces

The results of this study underscore the critical importance of water quality management, especially when integrating a significant proportion of ANC into fish feed. Specifically, replacing fishmeal with 20% ANC likely resulted in a decrease in the largest particle portion (>1000 μm), while the component of particles within the 250 to 1000 μm range increased. This shift may have implications for fecal removal efficiency as larger particles are generally easier to remove than smaller particles. Similar findings have been reported in previous studies, indicating that substituting fishmeal with plant ingredients can impact fecal quality. Brinker and Friedrich (2012) reported that a plant-based diet compromises fecal stability by generating more small particles compared to a fishmeal-based diet. Additionally, Schumann et al. (2022) observed that a complete replacement of fishmeal with plant proteins led to the rapid disintegration of feces from rainbow trout into very fine solids. This link between dietary ingredients or formulation and fecal quality highlights the importance of considering the impact on water quality management practices when evaluating alternative ingredients. Furthermore, the high moisture content in feces from the ANC-20 group also suggests poor water stability for those feces and underscores the need to consider water quality management when employing a high level of ANC in aquatic feed.

### 4.3 The effect of ANC on growth performance and nutrient utilization

A previous study by Olvera-Novoa et al., (1990) reported optimal weight gain, specific growth rate, feed intake, and nitrogen deposition in tilapia, *O. mossambicus*, when diets included alfalfa protein concentrates at levels ranging from 11.3% to 18.8%, replacing 15% to 20% of the fishmeal protein. However, inclusion levels ≥26.3% of alfalfa protein concentrates to replace 35% of fishmeal protein led to decreased performance compared to a control diet with 60% fishmeal. In our current study, significantly reduced growth performance was observed in fish fed diets containing 10% to 20% ANC to replace 12.5% to 25% fishmeal protein compared to those fed the control diet (ANC-0) formulated with 32% fishmeal. Tilapia as an omnivorous fish appears to have better tolerance to alfalfa protein compared to rainbow trout, a carnivorous fish. The documented advantage of omnivorous fish in utilizing plant ingredients over carnivorous fish supports this observation.

It's worth noting that besides species differences, the tilapia study used a higher fishmeal content (60% fishmeal) compared to our study (32% fishmeal). Therefore, comparisons regarding the percentage of fishmeal protein replacement between the two studies should be made with caution. Additionally, factors such as the quality of fishmeal, feed formulation, and feed processing methods could contribute to differing findings between the studies. These factors should be carefully considered in future research when testing this ingredient.

The reduced growth observed in trout fed 10% to 20% ANC can be partly attributed to the low digestibility of nutrients in these diets, as evidenced by a reduction in the digestibility of total dry matter and phosphorus. Phosphorus is an essential nutrient involved in various aspects of intermediary metabolism, playing a critical role in supporting fish growth and enhancing feed efficiency (Sugiura et al., 2004). In plant-based diets, phosphorus in the form of phytate is poorly available to fish due to the absence of phytase in their digestive tract and the poor utilization of phosphorus in its phytate form by fish has been extensively discussed in the literature (Hua and Bureau, 2006; Lall, 1991). Given that diet ANC-20 contained a higher level of phytate (0.24%) compared to diet ANC-0 (0.15%), we suggested that the phytate level should be considered as one of the factors contributing to the poor digestion of phosphorus in fish fed the ANC-based diets. Additionally, similar to other plant protein concentrates, the presence of indigestible carbohydrates, such as neutral detergent fiber (accounting for about 11% in ANC), may contribute to the observed reduction in dry matter digestibility in ANC-based diets (Reichert, 1981; Sinha et al., 2011). Protease inhibitors did not seem to be the primary contributors to the observed poor growth in our study, as we did not detect reduced protein digestibility with increasing ANC levels in the diet. Given that the digestibility of protein and most minerals were not negatively impaired by ANC-based diets, and in general, lipid digestibility is high, reduced digestibility of carbohydrates is likely another factor attributed to the overall low digestibility of dry matter in fish fed ANC-based diets. In addition, the ANC contained 2.7% crude fibers and 1.1% phytate, which might also impair the digestibility of nutrients. In contrast, the digestibility of trace minerals such as copper, iron, manganese, and zinc increased with the replacement of fishmeal by ANC, suggesting that ANC may serve as a good source of these trace minerals. This warrants further investigation in future studies.

Besides the reduced nutrient digestibility, an excess supply of iron may be another factor causing negative impacts on fish growth in the current study. The ANC-based diets had increased Fe levels, which was due to the high iron content in the ANC ingredient compared to fishmeal (1100 vs 550 mg/kg). Excess iron is toxic and can cause depressed growth, tissue damage, low nutrient utilization (Gatlin and Wilson, 1986), and even an increased mortality rate (Desjardins et al., 1987; Shiau and Su, 2003). This toxicity occurs as a result of the Fenton reaction, in which ferrous iron (Fe^2+^) is oxidized by the toxic hydroxyl radicals into ferric iron (Fe^3+^) (Winterbourn, 1995). A level of 274 mg Fe/kg diet had been reported to be optimal for rainbow trout fed a plant-based feed, but excess iron levels at 741 mg/kg diet were shown to depress fish growth and feed digestibility (Evliyaoğlu et al., 2022). Dietary Fe toxicity was also documented in rainbow trout fed levels higher than 1380 mg Fe/kg (Desjardins et al., 1987). Thus, we speculate that excess iron might be one of the factors associated with the reduced growth of rainbow trout when fed a high level of ANC-based diet. Furthermore, these levels may change depending on feed formulations, which may have different composition in terms of minerals, lipids, and proteins. A recent study by Wang et al. (2023) also discovered that the culture temperatures can impact the dietary requirement of iron l in spotted seabass with 178.5 and 209.0 mg/kg for fish reared at 27 and 33 °C, respectively. In addition, research is needed to investigate whether excess dietary iron may have an antagonistic effect on phosphorus availability.

Another potential factor contributing to the reduced growth of ANC-fed rainbow trout is the alteration of nutrient and energy metabolism influenced by ANC. This is supported by the alteration of metabolic pathways such as the citric acid cycle (tricarboxylic acid cycle or TCA), mitochondrial electron transport chain, ketone body metabolism, transfer of acetyl groups into mitochondria, in the fish fed ANC-20 diet when compared to the fish-fed ANC-0 diet. The liver is known to play a critical role in maintaining amino acid homeostasis except for branched-chain amino acids (BCAA). BCAA derived predominantly from muscle tissue can be degraded to form branched-chain ketoacids, which could then be converted back to BCAA by the liver. The increased levels of leucine and valine detected in the ANC-fed fish might be attributed to increased protein degradation from muscle and lower transaminase and ketoacid dehydrogenase activity in the liver compared to the muscle tissue (Suryawan et al., 1998). The reduced protein level in the fish fed ANC-20 diet further supports this interpretation. On the other hand, the amino acid threonine is both glucogenic and ketogenic and can be metabolized in the liver. The increased threonine could provide substrate to produce pyruvate and/or succinyl-CoA which in turn can enter the TCA cycle. This may explain, at least in part, the elevated levels of the TCA cycle intermediates, succinate and malate, detected in the liver of fish-fed ANC-based diet. Moreover, the alteration in the glucose-alanine cycle between fish fed the ANC-based diet and the ANC-0 diet agrees with the observation of reduced levels of alanine in the liver of rainbow trout fed the ANC-based diet. This suggests that alanine is used to synthesize glucose via the gluconeogenesis pathway or is converted into pyruvate and then enters the TCA cycle to be oxidized for energy. This observation is further supported by the increased levels of TCA intermediates observed in this study.

The primary role of betaine is as a methyl group donor in metabolism; however, this metabolite is also involved in osmotic pressure regulation as a known osmolyte, and it has been shown to possess antioxidant properties in fish tissues (Li et al., 2023; Virtanen et al., 1989). The reduced levels of betaine in fish fed the ANC-based diet could lead to changes in these biological functions. Betaine can be supplied through the diet or synthesized in the liver by oxidation of dietary choline. The reduced level of betaine in the ANC-fed fish may be due to the reduced content of fishmeal of those diets as fishmeal is one of the main sources of choline in the ANC-based diet. Supplementation of betaine or choline has been shown to improve lipid metabolism, reduce oxidation, and enhance liver health in animals, including fish fed low fishmeal diets (Eklund et al., 2005; Fan et al., 2014; Hansen et al., 2020; Jin et al., 2021; Li et al., 2023). However, despite lower betaine levels detected in ANC-20 fed fish, in our study no apparent deficiency symptoms were observed in the fish. The nutritional composition of the liver, hepatosomatic index, as well as blood and plasma chemistry parameters, were not significantly different in fish fed the ANC-diets when compared to the fish fed the ANC-0 diet, thus indicating that the dietary supply of betaine should be sufficient to maintain the fish normal performance under the current testing conditions. Long-term effects should be evaluated in future studies to test this hypothesis. Glycerol-3-P is a metabolite playing roles in lipid synthesis, glycolysis, and energy metabolism, which involves an electron transport chain to carry cytosolic reducing equivalents into the mitochondria for ATP synthesis. The increased level of glycerol-3-P in fish fed the ANC-20 diet suggests altered glycolipid metabolism as well as increased glycolysis in fish fed the ANC-based diet. This also aligns with the above discussion that inhibited glucose and glycogen synthesis occurred in the fish fed the ANC-based diet, as suggested by the reduced level of alanine.

### 4.4 Suggestions for future study

The decreased protein retention and protein efficiency suggest overall lower protein utilization for growth in ANC-fed fish. Although the levels of indispensable amino acids were similar across the current test diets, differences in their digestion and absorption may affect protein synthesis efficiency. While the digestibility coefficient for crude protein was similar among the test diets, individual amino acid digestibility was not measured, and this should be evaluated in future studies. A previous study by Larsen et al. (2012) reported delayed post-prandial amino acid absorption in fish fed a high plant-protein diet compared to a fishmeal control, likely due to the higher levels of indigestible carbohydrates in the plant-based diet. Brezas and Hardy (2020) found that enhanced coordination of amino acid availability contributed to the improved performance of rainbow trout selected for growth on plant protein based diets. Thus, improving the synchronization of amino acid absorption is suggested as a strategy to enhance plant protein synthesis. This warrants further research when ANC is incorporated into aquatic feed.

In the current study, the ANC-0 diet included 32% fishmeal, which is higher than the fishmeal level (10% to 20%) in practical feed (Biasato et al., 2022; Chemello et al., 2020). This control diet led to a 650% weight gain with an SGR of 3.2% after 9 weeks of feeding. Although the 20%-ANC diet contained only 20% fishmeal, the SGR and FCR (2.77% and 0.96, respectively) were comparable or superior to growth reported in previous studies (Fanizza et al., 2023; Lazzarotto et al., 2018; Roques et al., 2023). Furthermore, the nutrient composition and biochemical parameters of blood and plasma as well as feeding were not altered due to feeding diets with ANC levels up to 20% in the feed. These results suggest that the ANC-0 diet should contain an adequate or more than adequate nutrient supply as a control for comparing the two ingredients and provide fundamental information to guide future investigations on the application of ANC. Future studies, however, are needed to evaluate the potential of ANC in practical feed formulations with lower levels of fishmeal and compare its potential with other plant protein concentrates such as soy and corn protein. Furthermore, besides the nutritional quality of the product, sensory tests as well as production cost analysis, which were not addressed in this study, are critical for evaluating the potential of ANC used in commercial feed formulations.

## 5 Conclusion

In this pioneering investigation, we have conducted a comprehensive analysis of ANC utilization as an aquatic feed ingredient, integrating evaluations of feed management, feeding efficacy, and its impact on fish biology and metabolism, with a particular focus on rainbow trout. Our study reveals three pivotal findings based on the testing conditions:

1) Incorporating ANC into feed formulations enhances the physical quality of feed pellets, holding significant implications for feed management strategies.
2) Within the current feed formulation, up to 20% ANC inclusion does not have negative impacts on feeding behavior, nutritional quality based on proximate composition analysis, or manifest overt adverse clinical symptoms in rainbow trout.
3) ANC inclusion at 10% or higher levels obstructs nutrient digestibility, particularly affecting phosphorus and total dry matter, reduces protein utilization and alters fecal physical quality, consequently resulting in reduced fish growth.
4) Regarding fecal physical quality, the distribution of large particle sizes (>1000 μm) decreases, while the distribution of medium particles (250 to 1000 μm) increases with the inclusion of ANC, indicating that different management strategies may be needed to address this alteration.

Although no adverse effects on the proximate composition and clinical biochemical parameters were observed, metabolic shifts observed in fish fed with the ANC-20 diet warrant further investigation through long-term studies to validate our current findings. Moreover, our study underscores the necessity for additional research to explore the lasting impacts of ANC incorporation into fish feeds, particularly concerning nutrient utilization, overloading of iron content, fecal characteristics, and consequent water quality management in aquaculture settings. Prioritizing the optimization of phosphorus digestibility and achieving balanced amino acid profiles and their utilization emerges as imperative steps to maximize ANC utilization in aquatic feeds.

## CRediT authorship contribution statement

**Hu Chen:** Data curation, Formal analysis, Methodology, Writing – original draft. **Patrick C. Blaufuss:** Data curation, Writing – original draft. **Dong-Fang Deng:** Conceptualization, Data curation, Funding acquisition, Investigation, Methodology, Project administration, Resources, Writing – original draft, Writing – review & editing. **Fabio Casu:** Data curation, Methodology, Writing – original draft, Writing – review & editing. **Emma K. Kraco:** Data curation, Investigation, Methodology. **Brian Shepherd:** Conceptualization, Data curation, Funding acquisition, Investigation, Methodology, Resources. **Wendy M. Sealey:** Data curation, Formal analysis, Methodology, Writing – review & editing. **Aaron M. Watson:** Formal analysis, Methodology. **Matthew F. Digman:** Conceptualization, Funding acquisition, Writing – review & editing. **Deborah A. Samac:** Conceptualization, Funding acquisition, Writing – review & editing.

## Declaration of competing interest

We declare that we have no financial and personal relationships with other people or organizations that can inappropriately influence our work, and there is no professional or other personal interest of any nature or kind in any product, service and/or company that could be construed as influencing the content of this paper.

Mention of any trade names or commercial products in this article is solely for the purpose of providing specific information and does not imply recommendation or endorsement by the US Department of Agriculture. USDA is an equal opportunity provider and employer. Any mention of commercial products is to specify adequately the analytical procedures used. It does not imply recommendation or endorsement by NIST, or that the products mentioned are necessarily the best available for the intended purpose.
